# Unique and complementary suppression of cGAS-STING and RNA sensing- triggered innate immune responses by SARS-CoV-2 proteins

**DOI:** 10.1038/s41392-021-00515-5

**Published:** 2021-03-15

**Authors:** Yajuan Rui, Jiaming Su, Si Shen, Ying Hu, Dingbo Huang, Wenwen Zheng, Meng Lou, Yifei Shi, Meng Wang, Shiqi Chen, Na Zhao, Qi Dong, Yong Cai, Rongzhen Xu, Shu Zheng, Xiao-Fang Yu

**Affiliations:** 1 Cancer Institute, Second Affiliated Hospital, College of Medicine, Zhejiang University, Hangzhou, China; 2 School of Life Science, Jilin University, Changchun, China; 3 Department of Hematology, Second Affiliated Hospital, School of Medicine, Zhejiang University, Hangzhou, China

**Keywords:** Microbiology, Infectious diseases

## Abstract

The emergence of SARS-CoV-2 has resulted in the COVID-19 pandemic, leading to millions of infections and hundreds of thousands of human deaths. The efficient replication and population spread of SARS-CoV-2 indicates an effective evasion of human innate immune responses, although the viral proteins responsible for this immune evasion are not clear. In this study, we identified SARS-CoV-2 structural proteins, accessory proteins, and the main viral protease as potent inhibitors of host innate immune responses of distinct pathways. In particular, the main viral protease was a potent inhibitor of both the RLR and cGAS-STING pathways. Viral accessory protein ORF3a had the unique ability to inhibit STING, but not the RLR response. On the other hand, structural protein N was a unique RLR inhibitor. ORF3a bound STING in a unique fashion and blocked the nuclear accumulation of p65 to inhibit nuclear factor-κB signaling. 3CL of SARS-CoV-2 inhibited K63-ubiquitin modification of STING to disrupt the assembly of the STING functional complex and downstream signaling. Diverse vertebrate STINGs, including those from humans, mice, and chickens, could be inhibited by ORF3a and 3CL of SARS-CoV-2. The existence of more effective innate immune suppressors in pathogenic coronaviruses may allow them to replicate more efficiently in vivo. Since evasion of host innate immune responses is essential for the survival of all viruses, our study provides insights into the design of therapeutic agents against SARS-CoV-2.

## Introduction

A novel coronavirus, SARS-CoV-2, was identified in early 2020 as the cause of clusters of severe pneumonia in Wuhan, China.^1–3^ This novel severe disease was later designated COVID-19.^1–3^ SARS-CoV-2 shows high infectivity that has resulted in rapid global spreading. Currently, there is no vaccine or established therapy for COVID-19, and treatment is mainly based on supportive and symptomatic care.^4–6^ Therefore, the development of therapies that inhibit SARS-CoV-2 infection or replication are urgently needed.

It is believed that like other pathogenic coronaviruses such as SARS and MERS, SARS-CoV-2 is the result of cross-species transmission from bats to humans.^2^ The high transmissibility of SARS-CoV-2 in the human population has been attributed to an enhanced affinity of the SARS-CoV-2 spike protein for the human cellular receptor ACE2.^7–9^ The ability of SARS-CoV-2 to cause higher human-to-human spread and to readily infect multiple animal models^10–12^ indicates that SARS-CoV-2 has evolved to effectively antagonize multiple innate immune systems of its host. It is conceivable that SARS-CoV-2, as an RNA virus, needs to antagonize the RLR pathway. However, it is intriguing to consider that it may also be able to antagonize cGAS-STING activity, given that accumulating evidence indicates that RNA viruses^13^ can also be inhibited by cGAS-STING activation. This observation is particularly interesting because STING molecules in all the bats examined to date are defective for the induction of type-I interferon (IFN) and have compromised anti-viral activity.^14^ Defective STING activity could be one of the explanations for the persistence of viral infection, including that of coronaviruses, and the lack of disease manifestation in bats. Whether SARS-CoV-2 has maintained or regained the ability to inhibit STING during cross-species transmission is an open question.

In the current study, we evaluated the ability of SARS-CoV-2 structural and accessory proteins to antagonize innate immune responses by its host. In SARS-CoV-2, we observed complementary and unique patterns of viral protein-mediated suppression of distinct innate immune pathways. Diverse vertebrate STINGs, including those from humans, mice, and chickens, could be inhibited by ORF3a and 3CL of SARS-CoV-2. The innate immune suppression caused by SARS-CoV-2 proteins was found to be more effective than that caused by less pathogenic coronaviruses. These findings have implications for SARS-CoV-2 pathogenesis and reveal potential targets for the development of therapeutic strategies against SARS-CoV-2.

## Results

### Identification of SARS-CoV-2 proteins involved in innate immune suppression

**Fig. 1:**
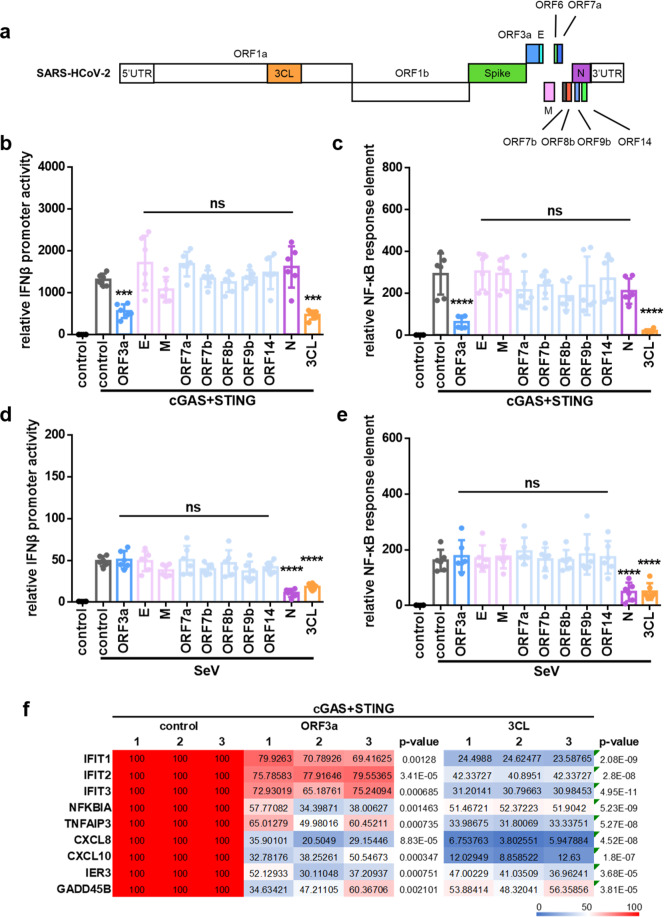
Identification of SARS-CoV-2 proteins involved in innate immune suppression. **a** Schematic diagram of the genomic organization and encoded proteins of SARS-2019 Wuhan-Hu-1 strain (MN908947.3). **b**, **c** cGAS-STING-stimulated IFNβ promoter (**b**) and NF-κB response element (**c**) activation is inhibited by ORF3a and 3CL. HEK293T cells were co-transfected with IFNβ-Luc (**b**), NF-κB-Luc (**c**), and the cGAS and STING expression vectors in the presence or absence of vectors expressing various SARS-CoV-2 proteins as indicated. **d**, **e** SeV-induced IFNβ promoter (**d**) and NF-κB response element (**e**) activation is inhibited by protein N and 3CL. HEK293T cells were co-transfected with IFNβ-Luc (**d**) or NF-κB-Luc (**e**) in the presence or absence of vectors expressing various SARS-CoV-2 proteins as indicated. SeV was added to the culture medium 12 h after transfection. pRL-TK Renilla was used as an internal control (**b**–**e**). Transactivation of the luciferase reporter was determined 24 h after transfection (**b**, **c**) or 12h after SeV infection (**d**, **e**) (*n* = 6 independent biological experiments). Means and standard deviations are presented. Statistical significance was determined by two-sided unpaired *t* test, ***p < 0.001; ****p < 0.0001; NS, no significance (**b–e**). **f** Inhibition of cGAS-STING-stimulated human genes by ORF3a and 3CL of SARS-CoV-2. HEK293T cells were co-transfected with the cGAS-STING expression vectors in the presence or absence of the ORF3a or 3CL expression vectors as indicated. Human genes activated by cGAS-STING alone served as a positive control and were set to 100%. Total RNA was prepared from HEK293T cells 24 h after transfection and analyzed for the transcriptional level of the indicated genes by RT-qPCR (*n* = 3 independent biological experiments). Statistical significance was determined by two-sided unpaired *t* test

During the early stages of RNA viral infection, the exposure of viral genomic RNA in the cytoplasm of infected cells is known to trigger the activation of the RLR pathway.^15–18^ Accumulating evidence indicates that RNA viral infection can also lead to the activation of STING because of viral–cellular membrane fusion and viral infection-induced mitochondrial damage.^19–21^ Viral structural proteins and virion-associated accessory proteins of SARS-CoV-2 (Fig. 1a) are more likely to be involved in the suppression of host innate immune responses during the initial stages of viral infection. Using established experimental systems for the detection of RLR- and cGAS-STING-triggered innate immune activation,^22–24^ we evaluated the innate immune modulation by various viral structural proteins, accessory proteins, and the main viral protease 3CL of SARS-CoV-2. We observed that 3CL had the ability to inhibit immune responses induced by both the RLR and cGAS-STING pathways (Fig. 1b–f and Supplementary Figs. 1b, c and 2e, f). SARS-CoV-2 structural protein N showed a strong ability to antagonize RLR innate immune activation (Fig. 1d, e), but had little effect on cGAS-STING-mediated innate immune activation (Fig. 1b, c). In contrast, SARS-CoV-2 ORF3a had the ability to specifically antagonize the immune activation induced by cGAS-STING, but no detectable effect on RLR-mediated immune responses (Fig. 1b–f).

### Characterization of SARS-CoV-2 proteins involved in cGAS-STING-mediated innate immune activation

**Fig. 2:**
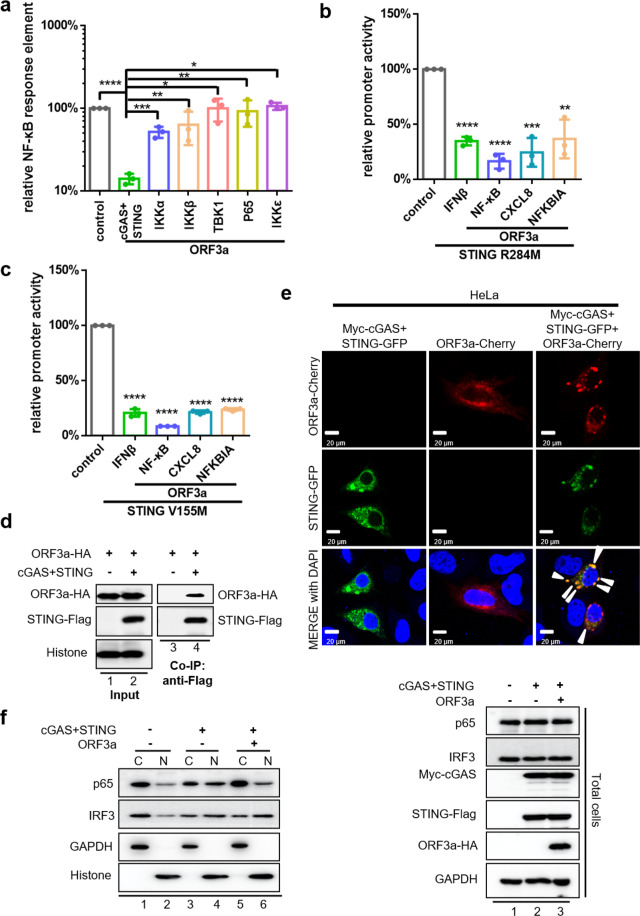
Characterization of ORF3a-mediated cGAS-STING inhibition. **a** Comparison of ORF3a-mediated inhibition of cGAS-STING-, IKKα-, IKKβ-, TBK1-, p65-, and IKKε-induced NF-κB signaling. HEK293T cells were co-transfected with NF-κB-Luc and the cGAS-STING, IKKα, IKKβ, TBK1, p65, or IKKε expression vectors in the presence or absence of the ORF3a expression vector. cGAS-STING, IKKα, IKKβ, TBK1, p65, or IKKε alone was set to 100%, as appropriate. **b**, **c** ORF3a inhibits the function of STING R284M (**b**) and STING V155M (**c**). HEK293T cells were transfected with IFNβ-Luc, NF-κB-Luc, CXCL8-Luc, or NFKBIA-Luc, together with STING R284M-Flag or STING V155M and empty vector or the ORF3a expression vector. Luciferase activity induced by STING R284M or STING V155M alone served as a control and was set to 100%. pRL-TK Renilla was used as an internal control (**a–c**). Transactivation of the luciferase reporter was determined 24 h after transfection (*n* = 3 independent biological experiments). Means and standard deviations are presented. Statistical significance was determined by two-sided unpaired *t* test, *p < 0.05; **p < 0.01; ***p < 0.001; ****p < 0.0001 (**a**–**c**). **d** Identification of the ORF3a–STING interaction by co-immunoprecipitation. HEK293T cells were transfected with ORF3a as well as Myc-cGAS and STING-Flag or control vector as indicated. Cell lysates were prepared and immunoprecipitated using anti-Flag beads 24 h after transfection. Precipitated samples were separated by SDS-PAGE, transferred to nitrocellulose membranes, and reacted with anti-HA antibody to detect ORF3a-HA and anti-Flag antibody to detect STING-Flag. Representative immunoblotting results are shown (*n* = 2 independent biological experiments). **e** Co-localization of intracellular ORF3a and STING. HeLa cells were transfected with Myc-cGAS and STING-GFP alone, ORF3a-Cherry alone, or Myc-cGAS and STING-GFP plus ORF3a-Cherry as shown. DAPI staining was performed to show the nucleus. Representative images from *n* = 3 independent biological experiments are shown. The arrowheads show representative co-localization of STING and ORF3a. **f** ORF3a inhibits nuclear translocation of p65, but not IRF3. HEK293T cells were transfected with the control vector or with STING-Flag and Myc-cGAS or with STING-Flag and Myc-cGAS plus ORF3a-HA, as indicated. Cells were harvested, the total cell lysates were prepared, and the nuclear (N) and cytoplasmic fractions (C) were separated 24 h after transfection. The indicated proteins were analyzed by immunoblotting using anti-p65 and anti-IRF3. GAPDH and histone were used as controls and detected using anti-GAPDH or anti-histone antibodies, respectively. Representative immunoblotting results are shown (*n* = 3 independent biological experiments)

**Fig. 3:**
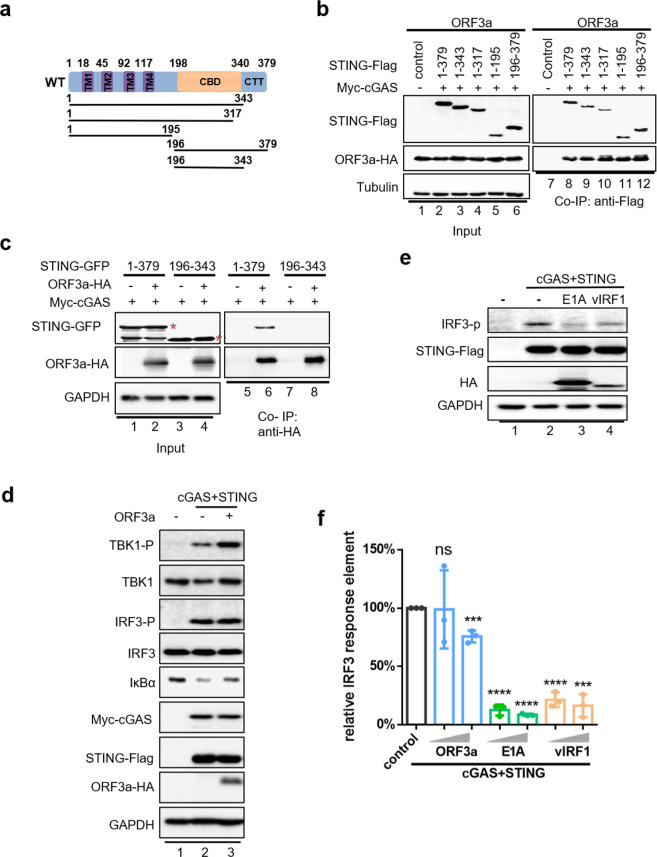
ORF3a interacts with both N- and C-terminal fragments of human STING, and has no effect on cGAS-STING triggered IRF3 activation. **a** Schematic of the STING functional domains and various STING truncation constructs. **b** Identification of the regions in STING that are important for the ORF3a interaction. HEK293T cells were co-transfected with the ORF3a expression vector and various STING mutant constructs or control vector. Cell lysates were prepared and immunoprecipitated using anti-Flag beads 24 h after transfection. Precipitated samples were separated by SDS-PAGE, transferred to nitrocellulose membranes, and reacted with anti-HA antibody to detect ORF3a-HA and anti-Flag antibody to detect STING truncation constructs. Tubulin was used as the loading control. **c** The N- and C-terminal of STING are required for the interaction with ORF3a. HEK293T cells were co-transfected with ORF3a expression vector and the wild-type or truncated form of STING. Cell lysates were prepared and immunoprecipitated using anti-HA beads 24 h after transfection. Precipitated samples were separated by SDS-PAGE, transferred to nitrocellulose membranes, and reacted with anti-GFP antibody to detect full-length STING or its truncation constructs and anti-HA antibody to detect ORF3a-HA. GAPDH was used as the loading control. The bands of STING and its truncation were labeled with an asterisk. **d** ORF3a impairs IκBα degradation induced by cGAS-STING, but has no effect on cGAS-STING-triggered IRF3 and TBK1 phosphorylation. HEK293T cells were co-transfected with STING-Flag, Myc-cGAS, and ORF3a-HA or control vector. After 24 h, total cells were harvested, and the protein expression levels were analyzed by immunoblotting with anti-TBK1-p, anti-TBK1, anti-IRF3-p, anti-IRF3, anti-IκBα, anti-Flag, anti-Myc, anti-HA, or anti-GAPDH antibodies**. e** E1A and vIRF1 inhibit the expression of cGAS-STING-induced IRF3 phosphorylation. HEK293T cells were co-transfected with STING-Flag, Myc-cGAS, E1A-HA, and vIRF1-HA or control vector. After 24 h, total cells were harvested, and the protein expression levels were analyzed by immunoblotting with anti-IRF3-p, anti-Flag, anti-HA, or anti-GAPDH antibodies. **f** E1A and vIRF1, but not ORF3a, inhibit IRF3 response element activity induced by cGAS-STING. HEK293T cells were co-transfected with IRF3-Luc, cGAS, and STING expression vectors, and increasing amounts of ORF3a, E1A, or vIRF1 expression vectors. Transactivation of the luciferase reporter was determined as described before (*n* = 3 independent biological experiments). Luciferase activity stimulated by cGAS-STING alone was used as a positive control and set to 100%. Means and standard deviations are presented. Statistical significance was determined by two-sided unpaired *t* test, ***p < 0.001; ****p < 0.0001; NS, no significance

Since modulation of cGAS-STING function by SARS-CoV-2 had not yet been reported, we characterized cGAS-STING suppression by SARS-CoV-2 proteins in greater detail. We found that viral ORF3a appears to have a preference for STING inhibition, because innate immune activation induced by cGAS-STING was suppressed to a greater extent than was suppression induced by downstream factors in the cGAS-STING pathway such as IKKα, IKKβ, TBK1, p65, and IKKε (Fig. 2a). Furthermore, ORF3a-mediated inhibition of cGAS-STING is likely due to the inhibition of STING, but not of cGAS, since ORF3a was able to inhibit the activity of STING activated by STING agonist (Supplementary Fig. 1d) or STING mutant R284M alone, in the absence of cGAS (Fig. 2b). In STING R284M, Arg284 is mutated to Met, causing constitutive, cGAMP-independent activation of STING.^25,26^ In addition, ORF3a was able to inhibit the function of the STING mutant V155M (Val to Met) in the absence of cGAS (Fig. 2c). This STING mutant also caused activation of STING that was independent of the constitutive activator cGAMP.^25,26^ ORF3a interacted with STING, as indicated by co-immunoprecipitation (co-IP) experiments (Fig. 2d). The interaction between ORF3a and STING was also confirmed by their intracellular co-localization, as revealed by immunofluorescence analysis (Fig. 2e). ORF3a could independently interact with the N-terminal fragment as well as the C-terminal fragment of STING (Fig. 3a, b), a finding that has not been reported for other viral proteins such as the E1A and vIRF1 proteins of DNA viruses.^22,27–30^ A truncated STING molecule missing both the N- and C-terminal regions lost the ability to interact with ORF3a (Fig. 3c).

We found that activation of cGAS-STING function induced a nuclear accumulation of nuclear factor-κB (NF-κB) factor p65 (Fig. 2f, compare lane 4 to lane 2, left panel; Fig. S5); this accumulation was inhibited by ORF3a (Fig. 2f, compare lane 6 to lane 4, left panel; Supplementary Fig. 5). This result is consistent with our observation of ORF3a-mediated suppression of NF-κB response element activity (Fig. 1c) and NF-κB target gene expression (Fig. 1f). In contrast to the suppression of NF-κB signaling, cGAS-STING-triggered IRF3 activation and IRF3 nuclear translocation (Fig. 2f) were not affected by ORF3a. IRF3 and TBK1 phosphorylation induced by cGAS-STING was also not inhibited in the presence of ORF3a, while IκBα degradation mediated by cGAS-STING was inhibited by ORF3a (Fig. 3d). In addition, cGAS-STING-induced activation of IRF3 response element function was also not inhibited by ORF3a (Fig. 3f). The ability of ORF3a-mediated inhibition of IFNβ promoter function was impaired when the NF-κB-response element in the IFNβ promoter was deleted (Supplementary Fig. 1e), indicating that ORF3a-mediated IFNβ promoter inhibition was due to its effect on NF-κB inhibition. These data suggest that ORF3a selectively suppresses cGAS-STING-mediated downstream signaling. This property of ORF3a is clearly different from that of the E1A and vIRF1 proteins of DNA viruses (Fig. 3e, f).

ORF3a is present in the genomes of the pathogenic coronaviruses SARS and SARS-CoV-2, but is missing from the genomes of the beta-coronaviruses HKU1 and OC43 (Supplementary Fig. 1a). The ORF4 sequence of HKU1 and the ORF5 sequence of OC43, located in similar positions in the viral genome, are very different from the ORF3a sequence of SARS-CoV-2 (Supplementary Fig. 1f). The amino acid sequences of ORF3a from SARS and SARS-CoV-2 have a high identity (Supplementary Fig. 1f), and ORF3a from SARS was also able to inhibit cGAS-STING function (Supplementary Fig. 1g). SARS-CoV-2 ORF3a has a Cys-rich region that has the potential to bind zinc. Interestingly, we found that *N*, *N*, *N*′, *N*′-tetrakis (2-pyridylmethyl)-ethylenediamine, a lipid-soluble zinc metal chelator, was able to block ORF3a function (Supplementary Fig. 1h).

**Fig. 4:**
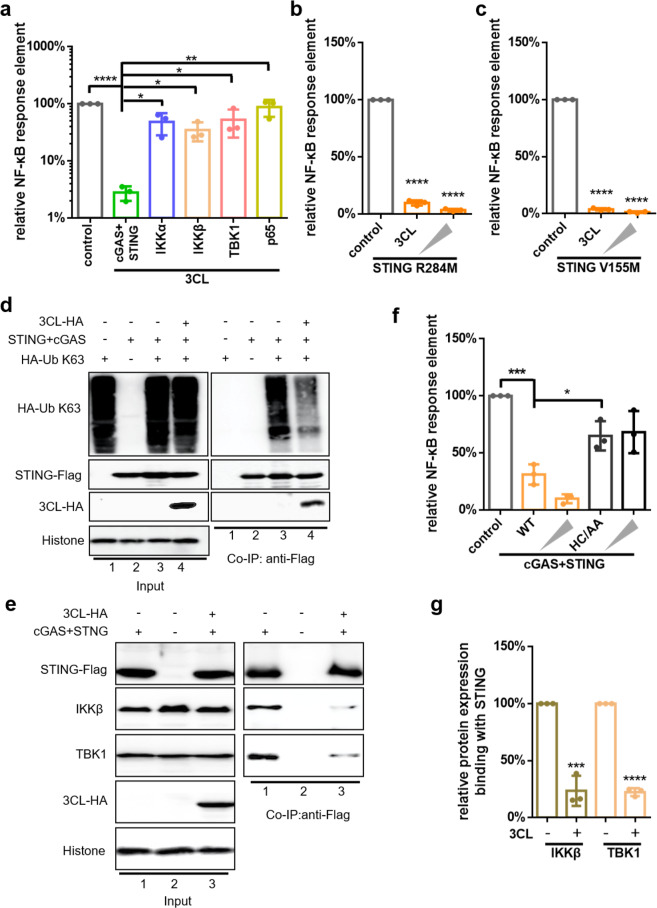
SARS-CoV-2 3CL selectively targets STING function and inhibits K63-Ub modification of STING. **a** SARS-CoV-2 3CL specifically antagonizes cGAS-STING-mediated NF-κB signaling. HEK293T cells were co-transfection with NF-κB-Luc and expression plasmids for cGAS and STING, IKKβ, IKKα, TBK1, or p65 with or without the expression vector for SARS-CoV-2 3CL. Luciferase activity stimulated by cGAS and STING, IKKβ, IKKα, TBK1, or p65 in the absence of SARS-CoV-2 3CL was set to 100%. **b**, **c** SARS-CoV-2 3CL inhibits STING R284M- (**b**) or V155M (**c**)-stimulated NF-κB signaling. HEK293T cells were transfected with NF-κB-Luc, pRL-TK Renilla, and the STING R284M (**b**) or V155M (**c**) expression vectors with increasing amounts of 3CL. **d** 3CL inhibits K63-Ub modification of STING. HEK293T cells were transfected with Ub K63 alone, STING-cGAS alone, or co-transfected with STING, cGAS, and Ub K63 in the presence or absence of 3CL. MG132 (20 µM) was added 12 h later. Cell lysates were prepared and immunoprecipitated with anti-Flag beads 24 h after transfection. Precipitated samples were prepared and reacted with anti-Flag antibody to detect Flag-STING and anti-HA antibody to detect HA-Ub-K63 and 3CL-HA. Histone was used as the loading control. **e** 3CL disrupts the interaction between STING and endogenous TBK1 or IKKβ. HEK293T cells were transfected with STING-cGAS in the presence or absence of 3CL. Cell lysates were prepared and immunoprecipitated using anti-Flag beads 24 h after transfection. Precipitated samples were prepared and reacted with anti-Flag antibody to detect STING-Flag and anti-TBK1 and anti-IKKβ antibody to detect endogenous proteins. Histone was used as the loading control. **f** The 3CL mutant HC/AA has a reduced ability to inhibit cGAS-STING-stimulated NF-κB response element activation. HEK293T cells were transfected with NF-κB-Luc, pRL-TK Renilla, and the cGAS-STING expression vector with increasing amounts of 3CL or 3CL HC/AA. cGAS-STING alone served as a positive control and was set to 100%. Transactivation of the luciferase reporter was determined 24 h after transfection (*n* ≥ 3 independent biological experiments) (**a**–**c**, **f**). **g** The binding ability of IKKβ or TBK1 with STING was downregulated in the presence of 3CL in (**e**) and was quantified using the ImageJ software. Means and standard deviations are presented. Statistical significance was determined by two-sided unpaired *t* test, *p < 0.05; **p < 0.01; ***p < 0.001; ****p < 0.0001 (**a**–**c**, **f**–**g**)

SARS-CoV-2 3CL is also a potent inhibitor of cGAS-STING function (Fig. 1b, c, f). To further evaluate the target of 3CL-mediated cGAS-STING inhibition, we first compared the ability of 3CL to inhibit the immune activation induced by cGAS-STING and by downstream factors. The ability of cGAS-STING to trigger NF-κB signaling was suppressed more effectively by 3CL than was that triggered by IKKα, IKKβ, TBK1, or p65 (Fig. 4a). We have confirmed 3CL-mediated cGAS-STING inhibition in human lung cell line A549 (Supplementary Fig. 6a) and human Huh-7 cell line (Supplementary Fig. 6b), which have frequently been used for SARS-CoV-2 study.^31^ Similar results were also obtained for SARS-CoV-2 ORF3a (Supplementary Fig. 6a, b). Furthermore, in the absence of cGAS, STING R284M- and STING V155M-mediated immune activation could still be efficiently suppressed by 3CL (Fig. 4b, c and Supplementary Fig. 2a, b), suggesting that 3CL does not target cGAS. Similar to ORF3a, 3CL also suppressed cGAS-STING-mediated p65 nuclear accumulation (Supplementary Figs. 2d and 5), but had no effect on IRF3 nuclear translocation (Supplementary Fig. 2d). STING-mediated NF-κB signaling requires K63-ubiquitin-mediated modification of STING,^32^ a modification that is critical for downstream protein binding.^32,33^ 3CL bound to STING and specifically inhibited this modification (Fig. 4d). Moreover, the recruitment of TBK1 and IKKβ by STING was inhibited by SARS-CoV-2 3CL (Fig. 4e, g). K63 ubiquitination is a classic modification of innate immune pathways.^33–35^ However, unlike that involving STING, K63-ubiquitin modification of other innate immune key factors such as MAVS, MYD88, or TRAF3 was not affected by 3CL (Supplementary Fig. 3a–c). In contrast to cGAS-STING, MAVS- and MYD88-triggered NF-κB signaling were also not inhibited by 3CL (Supplementary Fig. 3d, e). The enzymatic active site of 3CL appears to be important, since a 3CL mutant (HC/AA)^36^ containing amino acids H41 and C145 substituted with AA had a reduced ability to inhibit cGAS-STING function (Fig. 4f and Supplementary Figs. 2c and 4a). 3CL HC/AA was also less effective than 3CL in inhibiting the K63-ubiquitin-mediated STING modification (Supplementary Fig. 4b).

### SARS-CoV-2 proteins exhibit inhibitory activity against diverse vertebrate STING molecules

**Fig. 5:**
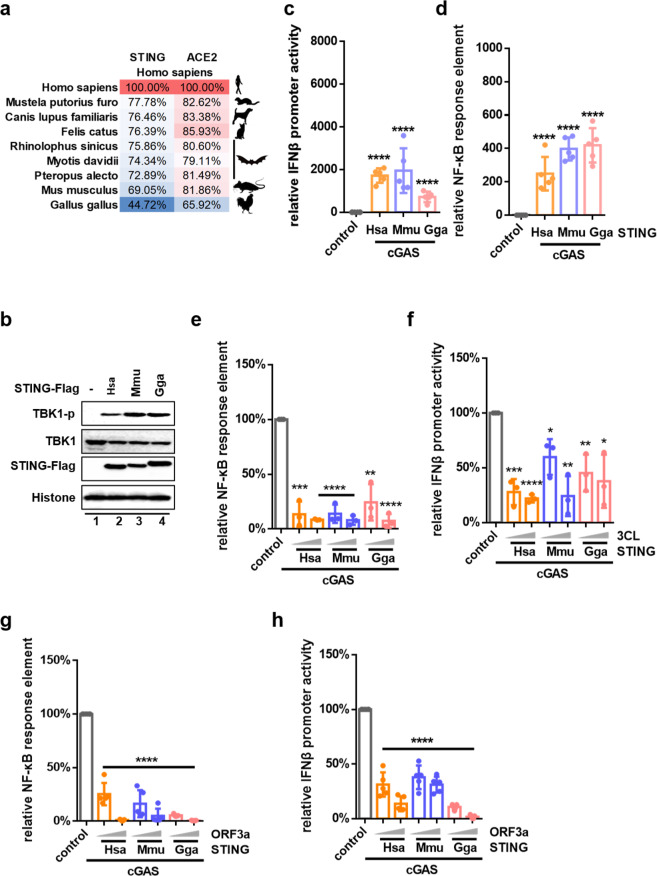
Human, mouse, and chicken STING function can be suppressed by SARS-CoV-2 3CL. **a** Percentage of amino acid identity of representative vertebrate STING molecules as compared to *Homo sapiens* STING [*Homo sapiens* (NP_938023.1), *Felis catus* (AMD40259.1), *Canis lupus familiaris* (XP_022263973.1), *Mustela putorius furo* (XP_012907883.1), *Myotis davidii* (XP_006772500.1), *Pteropus alecto* (XP_006923104.1), *Rhinolophus sinicus* (XP_019595754.1), *Mus musculus* (NP_082537.1), and *Gallus gallus* (NP_001292081.1)]. Identities of human ACE2 with representative vertebrate ACE2 are shown for comparison. **b** *Homo sapiens*, *Mus musculus*, and *Gallus gallus* STING molecules can promote TBK1 phosphorylation in human cells. HEK293T cells were co-transfected with the cGAS and *Homo sapiens*, *Mus musculus*, or *Gallus gallus* STING expression vectors, respectively. Cells were harvested 24 h after transfection, and the protein expression levels were analyzed by immunoblotting with anti-Flag, anti-TBK1, anti-TBK1-p, or anti-Histone antibody. **c**, **d** *Homo sapiens*, *Mus musculus*, and *Gallus gallus* STING can all stimulate IFNβ (**c**) promoter and NF-κB- (**d**) response element activity. HEK293T cells were transfected with IFNβ-Luc (**c**) or NF-κB-Luc (**d**) and pRL-TK Renilla, together with Myc-cGAS and each of the three vertebrate STING molecules described previously. **e–h** SARS-CoV-2 3CL (**e**, **f**) and ORF3a (**g**, **h**) inhibit vertebrate STING function in a dose-dependent manner. HEK293T cells were transfected with NF-κB-Luc (**e**, **g**) or IFNβ-Luc (**f**, **h**) and pRL-TK Renilla, together with STING-Flag and Myc-cGAS and increasing amounts of the 3CL (**e**, **f**) or ORF3a (**g**, **h**) expression vectors. Luciferase activity induced by cGAS-STING alone was used as a positive control and set to 100%. Transactivation of the luciferase reporter was determined 24 h after transfection (*n* ≥ 3 independent biological experiments). Means and standard deviations are presented. The statistical significance analyses were performed using a two-sided unpaired *t* test, *p < 0.05; **p < 0.01; ***p < 0.001; ****p < 0.0001

SARS-CoV-2 can infect a wide range of mammals. The amino acid sequences of mammalian STING molecules are quite divergent (Fig. 5a), and mouse and chicken STING molecules are even more divergent (Fig. 5a), but they all were able to induce efficient innate immune responses in human cells, as indicated by an induction of TBK1 phosphorylation (Fig. 5b) and activation of IFNβ promoter (Fig. 5c) and NF-κB-response element activity (Fig. 5d). We observed that SARS-CoV-2 3CL could efficiently inhibit the function of human, mouse, and chicken STING (Fig. 5e, f and Supplementary Fig. 6e, f). These results suggest that SARS-CoV-2 3CL may target a conserved feature of vertebrate STING molecules. Similarly, SARS-CoV-2 ORF3a was also able to inhibit the function of human, mouse, and chicken STING (Fig. 5g, h and Supplementary Fig. 6c, d).

## Discussion

A unique pathogenic feature of COVID-19 is a heightened suppression of innate immunity and elevated inflammatory responses.^37^ Innate immune suppression by SARS-CoV-2 is poorly understood. We have now identified several SARS-CoV-2 viral proteins that differentially target distinct human innate immune responses. These viral proteins have complementary activity toward different innate immune pathways (Supplementary Fig. 7). We found that ORF3a selectively inhibited cGAS-STING function, but was ineffective against the RLR pathway. ORF3a is present in the genomes of the pathogenic coronaviruses SARS-CoV-2 and SARS, but not in those of the less pathogenic beta-coronaviruses HKU1 or OC43, which also infect humans (Supplementary Fig. 1a).

In contrast to SARS-CoV-2 ORF3a’s selective inhibition of cGAS-STING activity and lack of effectiveness against the RLR pathway, structural protein N of SARS-CoV-2 had strong inhibitory activity toward the RLR pathway, but had little inhibitory effect against cGAS-STING function (Fig. 1b–e). Recently, SARS-CoV-2 NSP13, NSP14, NSP15, and ORF6 have been reported to inhibit RLR pathway.^38,39^ Nsp1 prevents translation of IFNs IFN-β and IFN-λ1 and interleukin-8, as well as IFN-stimulated anti-viral factors through binding to the human ribosomal machinery.^38,40^ Direct cleavage of IRF3 by SARS-CoV-2 NSP3 has also been observed.^41^ The presence of multiple efficient immune-suppressive viral proteins in SARS-CoV-2 and SARS may contribute to their pathogenic potential in humans.

We also discovered that in SARS-CoV-2, 3CL (the main protease of coronaviruses) played a major role in the suppression of host innate immune responses triggered by the cGAS-STING and RLR pathways (Figs. 1b–f, 4a–c, f and Supplementary Fig. 2a–c, e, f); in coronaviruses, this protease is responsible for the cleavage of multiple ORF1a and ORF1b polyproteins and is essential for viral replication.^42,43^

It is interesting to note that SARS-CoV-2 uses at least two viral proteins to antagonize cGAS-STING activity. Accumulating evidence indicates that RNA viruses,^13^ in general, can trigger cGAS-STING responses and be inhibited by cGAS-STING activation. Bats are believed to be a natural host for many coronaviruses,^2,44^ and STING molecules in all the bats examined to date are defective for the induction of type-I IFN and have compromised anti-viral activity.^14^ This defective STING activity has been linked to the persistence of coronavirus infection in bats. Apparently, SARS-CoV-2 has maintained or regained the ability to inhibit STING function during cross-species transmission. Both 3CL and ORF3a of SARS-CoV-2 can antagonize various vertebrate STING function such as those from humans, mice, and chickens, consistent with the observations that SARS-CoV-2 can infect a wide range of animals (Fig. 5e–h and Supplementary Fig. 6c–f).

Host innate immune responses are potent barriers to the cross-species transmission of viruses.^44–46^ Consequently, all viruses have developed efficient mechanisms for evading host innate immune responses in order to survive. The existence of several SARS-CoV-2 proteins that simultaneously antagonize distinct human innate immune pathways indicates the importance of host defense against SARS-CoV-2. Future work should explore ways to develop therapeutic strategies based on SARS-CoV-2 viral protein-mediated evasion of innate immune responses as drug targets.

## Materials and methods

### Cell line, viruses, plasmids, and antibodies

A549 cells (ATCC, CCL-185), Huh-7 cells (DSMZ, BFN60800691), HEK293T cells (ATCC, CRL-3216), and HeLa cells (ATCC, CCL-2) were cultured in Dulbecco’s modified Eagle’s medium supplemented with 10% fetal bovine serum and 1% penicillin/streptomycin solution. All the coronavirus protein constructs utilized in this study were obtained from Generay Biotech Co., Ltd, China, including N, M, E, ORF3a, ORF7a, ORF7b, ORF8b, ORF9b, ORF14, 3CL, and 3CL HC/AA from SARS-2 (MN908947.3) and ORF3a from SARS (AY278741.1). In brief, fragments containing the coding sequences of CoV proteins flanked by 5′ *Sal*I and 3′ *Bam*HI sites were inserted into the VR1012 vector with a C-terminal HA tag. pRK5-HA-Ubiquitin-K63 (#17606) was obtained from Addgene. The CXCL8 promoter (−271 to +62 bp) luciferase reporter plasmid and NFKBIA promoter (−360 to +5 bp) luciferase reporter plasmid were amplified from the genomic DNA of HEK293T cells and cloned into the PGL3 basic vector using the *Kpn*I and *Bgl*II sites. ORF3a-Cherry sequence was amplified from SARS-CoV-2 ORF3a and inserted into the pmCherry-N1 vector. Myc-cGAS, STING-Flag, STING-Flag truncations, MAVS-Flag, IKKβ-Flag, IKKα-Flag, TBK1-Flag, p65-Flag, IKKε-Flag, MDA5-CARD-Flag, and other luciferase reporter plasmids were described previously.^22,23^ Sendai virus (20 HA/mL) was prepared as previously described.^23^ *Mus musculus* STING (MF622063.1), *Gallus gallus* STING (KP893157.1), MYD88, and TRAF3 were synthesized and inserted into VR1012 vector with a C-terminal Flag tag and E1A (NC_001405.1) and vIRF1 (NC_009333.1) were synthesized and inserted into VR1012 vector with a C-terminal HA tag by Generay Biotech Co., Ltd, China. P65-GFP was gifted by Dr. Fengyi Wan^47^ (Department of Biochemistry and Molecular Biology, Johns Hopkins University). STING-GFP was gifted from the Laboratory of K. Fitzgerald (University of Massachusetts Medical School). STING agonist‐3 (≥99.94% purity) (#HY‐103665) was acquired from MCE (Shanghai, China). The antibodies utilized in this study are outlined in Table S1.

### Immunoblotting and co-IP

Cell samples were harvested, then washed twice with cold phosphate-buffered saline (PBS), and lysed in lysis buffer (50 mM Tris, pH 7.5, with 150 mM NaCl, 1% NP-40, 0.5% deoxycholate sodium, 9 mM EDTA, and complete protease inhibitor cocktail tablets [Roche, USA]) at 4 °C for 30 min, and then mixed into 1× loading buffer (0.08 M Tris, pH 6.8, with 2.0% sodium dodecyl sulfate (SDS), 10% glycerol, 0.1 M dithiothreitol, and 0.2% bromophenol blue) and boiled for 20 min. Cell lysates were separated on SDS-polyacrylamide gel electrophoresis (SDS-PAGE) gels and transferred to nitrocellulose membranes using a semidry apparatus (Bio-Rad, USA). Membranes were probed overnight with various primary antibodies against the proteins of interest. Secondary antibodies were horseradish peroxidase-conjugated anti-rabbit IgG (HuaBio, HA1006) and anti-mouse IgG (HuaBio, HA1001). Staining was performed with ECL Substrate (Proteintech, USA) according to the manufacturer’s protocol. HEK293T cells were seeded onto 6-cm dishes and transfected with a total of 3 μg of empty plasmid or various expression plasmids using Hieff Trans™ Liposomal Transfection Reagent (YeasenBiotech, China). At 36 h after transfection, the cells were lysed in lysis buffer (50 mM Tris, pH 7.5, with 150 mM NaCl, 0.5% NP-40, and complete protease inhibitor cocktail tablets [Roche, SUI]) at 4 °C for 30 min. After sonication and centrifugation, the cell lysates were incubated with Anti-Flag Affinity Matrix (Sigma A-2220, USA) or Anti-HA Affinity Matrix (Roche, SUI) at 4 °C overnight on a rotator. After six washes with wash buffer (20 mM Tris, pH 7.5, 100 mM NaCl, 0.05% Tween-20, 0.1 mM EDTA), 50 μL of elution buffer (100 mM glycine-HCl, pH 2.5) was added to resuspend the beads, and the eluted proteins were obtained by centrifugation, followed by SDS-PAGE and immunoblotting analysis. Immunoblots were developed with Super ECL Detection Reagent (YeasenBiotech, China) chemiluminescent substrates. Membranes were exposed to films or imaged with a Series c300 (Azure Biosystems, USA).

### RNA extraction and quantitative real-time PCR (RT-qPCR)

Total RNA from cells was isolated using Trizol (Life Technologies, USA) according to the manufacturer’s instructions, including the DNase I digestion step. Samples were incubated in 10 μL of diethyl pyrocarbonate (DEPC)-treated water with 1× RQ1 RNase-Free DNase buffer, 1 μL of RQ1 RNase-Free DNase (Promega, USA), and 4 U of RNase inhibitor (New England Biolabs) for 30 min at 37 °C. The DNase activity was inactivated by the addition of 1 μL RQ1 DNase stop solution and incubation at 65 °C for 10 min. The RNA was reverse-transcribed by using random primers and the Multiscribe Reverse Transcriptase from the High-Capacity cDNA Archive Kit (Applied Biosystems, USA) according to the manufacturer’s instructions. The complementary DNA was either undiluted or serially diluted in DEPC-treated water before the real-time PCR reaction to ensure that the amplification was within the linear range of detection. The StepOne Real-Time PCR system (Applied Biosystems, USA) and LightCycler480 (Roche, SUI) were used for the RT-qPCR amplifications. The reactions were performed under the following conditions: 95 °C for 30 s, followed by 40 cycles of 95 °C for 5 s and 60 °C for 34 s, followed by a dissociation protocol. Single peaks in the melting-curve analysis indicated specific amplicons. The target sequences were amplified using primer pairs. qPCR primers are listed in Table S2.

### Luciferase assays

HEK293T, A549, or Huh-7 cells were plated onto 24-well dishes and transfected the following day as indicated: 100 ng of the reporter plasmid for IRF3- and NF-κB-response elements, IFNβ, CXCL8, or IKBA promoter, 5 ng Renilla luciferase control plasmid (pRL-TK), and the indicated amounts of the expression plasmids were used per well. Fifty nanograms of SARS-CoV-2 proteins were transfected in the luciferase reporter assay. Fifty and one hundred fifty nanograms of 3a or 3CL was transfected in Figs. 3f, 4b, c, f, 5e–h and Figs. S1g, S4, and S6. At 24 h after transfection, the transactivation activity of the promoters was determined by measuring firefly and Renilla luciferase activities using the Dual-Luciferase Reporter Assay Kit (catalog no. E2810; Promega, USA). The expression of proteins was confirmed by immunoblotting. Firefly luciferase activity was normalized to Renilla luciferase.

### Nuclear and cytoplasmic fractionation

HEK293T cells were transfected with the indicated plasmids for 36 h and then divided into two equal portions: one portion was loaded in 120 μL 1× loading buffer to confirm the level of protein expression (whole-cell lysate), and the other was prepared by scraping HEK293T cells into cold PBS and processed using Thermo Scientific™ NE-PER™ Nuclear and Cytoplasmic Extraction Reagents (catalog no. 78833; Thermo Scientific, USA) according to the manufacturer’s protocol.

### Confocal microscopy

HeLa and HEK293T cells grown on coverslips were fixed for 15 min with 4% paraformaldehyde in PBS, and then blocked using 5% bovine serum albumin for 1 h. The nuclei were counterstained with 4′,6-diamidino-2-phenylindole (Sigma-Aldrich, USA). Images were captured using a ZEISS laser scanning confocal microscope (Zeiss LSM 710). A ZEISS ZEN Microscope software was used for acquisition.

### Statistical analysis

Data from the luciferase reporter assays and RT-qRCR results are presented as means and standard errors, generated using the GraphPad Prism software, version 6.01 (GraphPad Software Inc., 2007). The statistical significance analyses were performed using two-sided unpaired *t* test (*P* values). NS, no significance, *p < 0.05, **p < 0.01, ***p < 0.001, and ****p < 0.0001).

## Supplementary information

Supplementary Materials

Supplementary tables
