# Supplementary material for: Unique and complementary suppression of cGAS-STING and RNA sensing- triggered innate immune responses by SARS-CoV-2 proteins
Source: Signal Transduct Target Ther. 2021 Mar 15;6:123. doi: 10.1038/s41392-021-00515-5 (PMC7958565; doi:10.1038/s41392-021-00515-5)
Supplement: Supplementary file 1 — Supplementary Materials [file 41392_2021_515_MOESM1_ESM.docx]

Supplementary Materials for

Unique and complementary suppression of cGAS-STING and RNA sensing- triggered innate immune responses by SARS-CoV-2 proteins

Yajuan Rui^1,4^, Jiaming Su^1,4^, Si Shen^1,4^, Ying Hu^1^, Dingbo Huang^1^, Wenwen Zheng^1^, Meng Lou^1^, Yifei Shi^1^, Meng Wang^1^, Shiqi Chen^1^，Na Zhao^1^, Qi Dong^1^, Yong Cai^2^, Rongzhen Xu^3^, Shu Zheng^1^, Xiao-Fang Yu^1^*

^1^Cancer Institute, Second Affiliated Hospital, College of Medicine, Zhejiang University, Hangzhou, 310009, China

^2^School of Life Science, Jilin University, Changchun,130061, China

^3^Department of Hematology, Second Affiliated Hospital, School of Medicine, Zhejiang University, Hangzhou, 310009, China

^4^These authors contributed equally：Yajuan Rui, Jiaming Su, Si Shen.

*Correspondence: [xfyu1@zju.edu.cn](mailto:xfyu1@zju.edu.cn)

**This PDF file includes:**

Figures. S1 to S7

Supplementary Tables S1 to S2

Figure. S1.

**
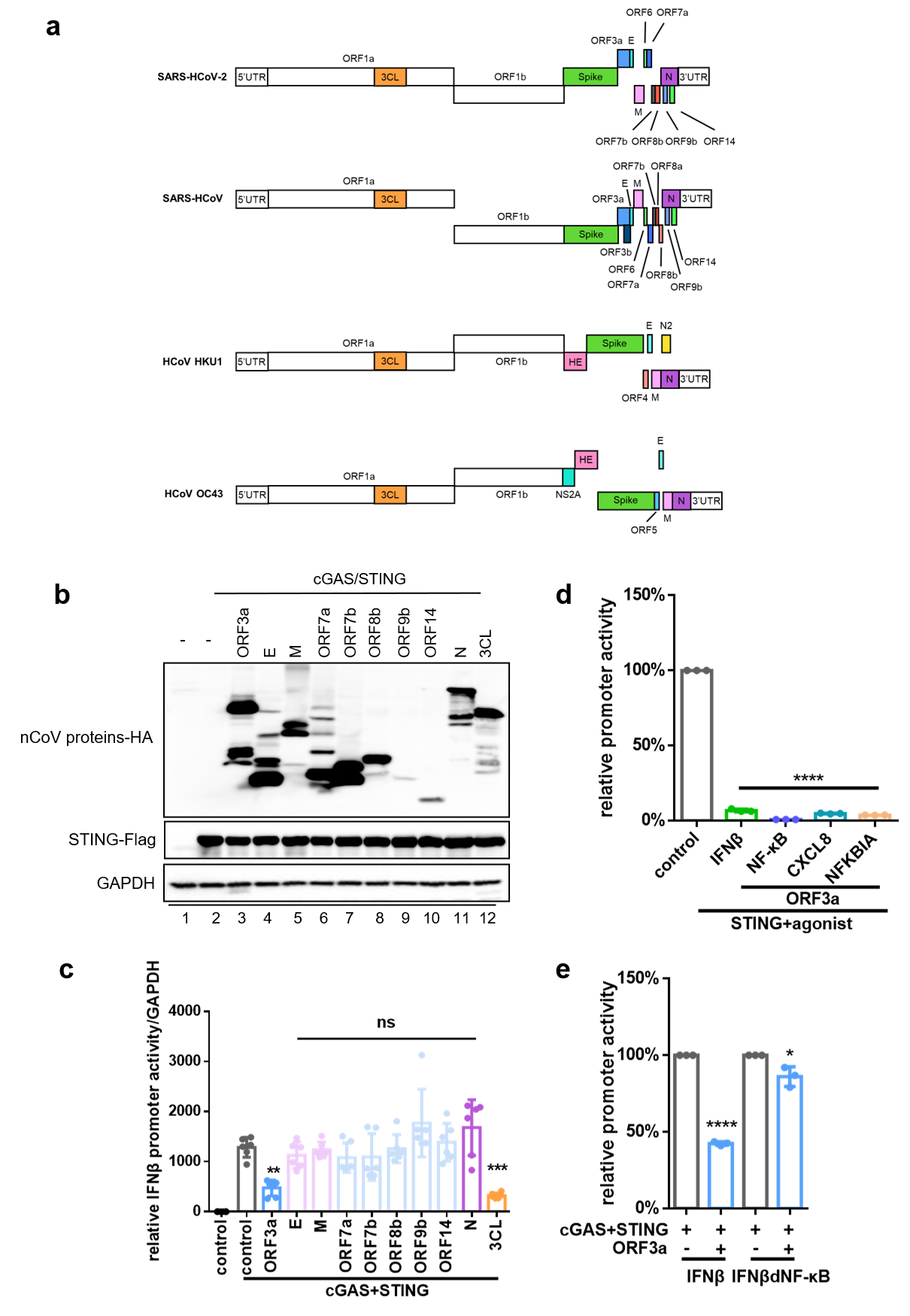
**

**
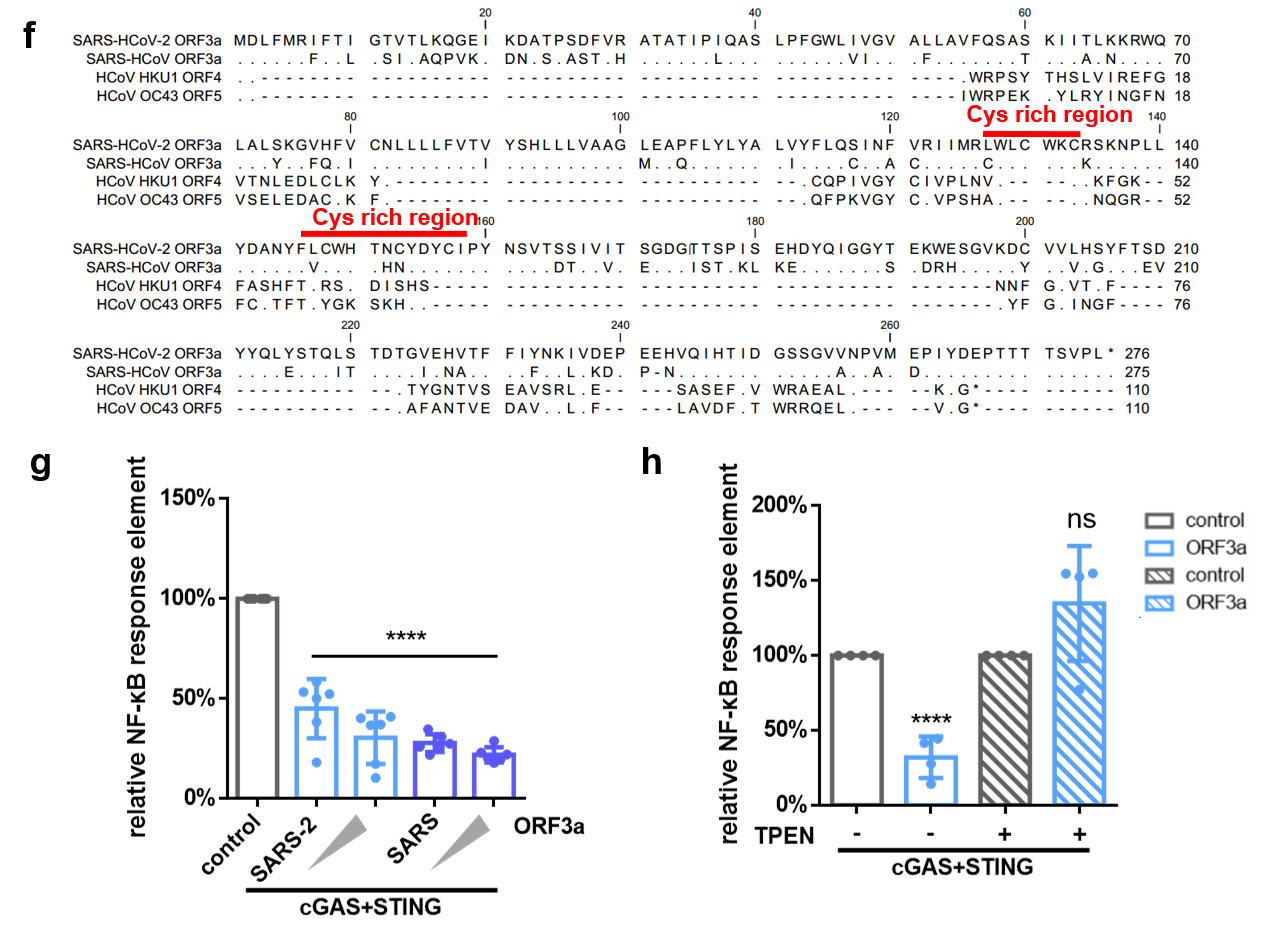
Fig. S1 a,** Schematic diagram of the genomic organization of β-coronavirus family members SARS-HCoV-2 (MN908947.3), SARS-HCoV(AY278741.1), HCoV HKU1(NC_006577.2), and HCoV OC43(NC_006213.1). **b,** total cell lysates were prepared from **(Fig. 1b)**, the expression level of SARS-CoV-2 proteins and STING expression was analyzed by immunoblotting using anti-HA and anti-Flag antibodies. GAPDH was used as an internal control. **c,** IFNβ promoter luciferase activity in **(Fig. 1b)** was normalized using GAPDH (n = 6 independent biological experiments). **d,** ORF3a inhibits the function of STING agonist-3. HEK293T cells were transfected with IFNβ-Luc, NF-κB-Luc, CXCL8-Luc, or NFKBIA-Luc, together with STING and empty vector or the ORF3a expression vector. STING agonist-3 (0.3 μM) was added to the culture medium 2 h after transfection. **e,** ORF3a inhibits cGAS-STING-stimulated IFNβ but has little effect on IFNβ△NF-κB promoter activation. HEK293T cells were transfected with IFNβ-Luc and IFNβ△NF-κB-Luc, together with cGAS, STING and empty vector or the ORF3a expression vector. **f,** Alignment of ORF3a sequences of SARS-HCoV-2 and SARS-HCoV, ORF4 of HCoV HKU1, and ORF5 of HCoV OC43. The Cys-rich regions of ORF3a are marked with red line. **g,** ORF3a from SARS-HCoV-2 and SARS-HCoV both have the ability to inhibit cGAS-STING function in a dose-dependent manner. HEK293T cells were co-transfected with NF-κB-Luc, the cGAS-STING expression vectors, and increasing amounts of SARS-CoV-2 or SARS-CoV ORF3a. **h,** TPEN can block the inhibition of ORF3a. HEK293T cells were co-transfected with NF-κB-Luc and the cGAS-STING expression vectors in the presence or absence of ORF3a. TPEN (3 µM) was added to the culture medium 12 h after transfection (columns with slash). pRL-TK Renilla was used as an internal control **(d-e, g-h)**. Transactivation of the luciferase reporter was determined 24 h after transfection (n ≥ 3 independent biological experiments). Luciferase activity stimulated by STING with STING agonist-3 **(d)** or cGAS-STING **(e, g-h)** was used as a positive control and set to 100%. Means and standard deviations are presented. Statistical significance was determined by two-sided unpaired t-test，*p<0.05; **** p<0.0001; NS, no significance **(c-e, g-h)**.

Figure. S2.


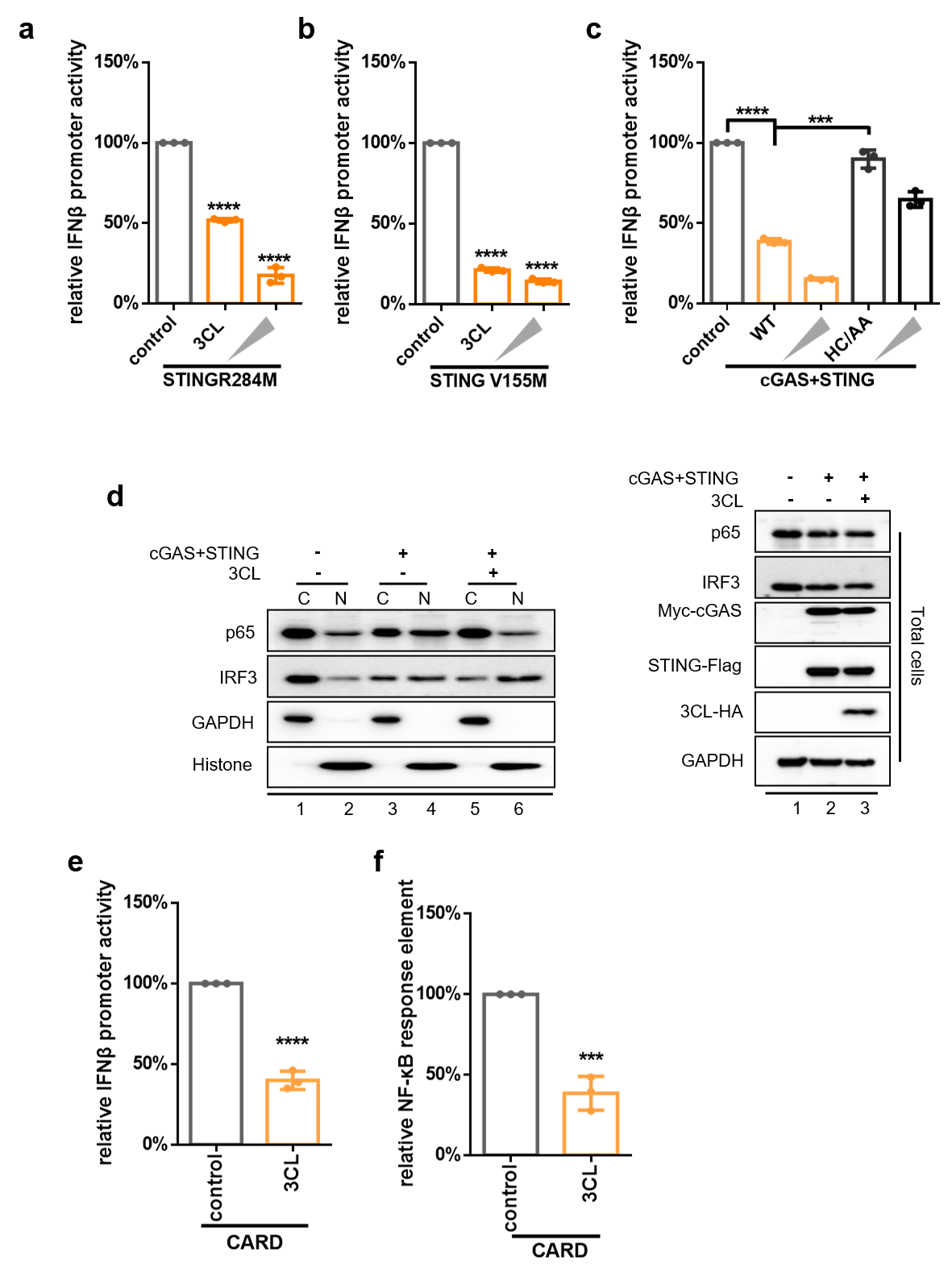


**Fig. S2 a-b**, SARS-CoV-2 3CL inhibits STING R284M **(a)** or V155M **(b)**-stimulated IFNβ promoter activation. HEK293T cells were transfected with IFNβ-Luc, pRL-TK Renilla, and the STING R284M**(a)** or V155M**(b)** expression vectors with increasing amounts of 3CL. **c,** The 3CL mutant HC/AA has a reduced ability to inhibit cGAS–STING-stimulated IFNβ promoter activation. HEK293T cells were transfected with IFNβ-Luc, pRL-TK Renilla, and the cGAS-STING expression vectors with increasing amounts of 3CL or 3CL HC/AA. cGAS-STING alone served as a positive control and was set to 100%. **d,** 3CL inhibits nuclear translocation of p65 but not IRF3. HEK293T cells were transfected with the control vector or with STING-Flag and Myc-cGAS or with STING-Flag and Myc-cGAS plus 3CL-HA, as indicated. Cells were harvested, the total cell lysates were prepared and the nuclear (N) and cytoplasmic fractions (C) were separated 24 h after transfection. The indicated proteins were analyzed by immunoblotting using anti-p65 and anti-IRF3 antibodies. GAPDH and Histone were used as controls and detected using anti-GAPDH or anti-Histone antibodies, respectively. Representative immunoblotting results are shown (n=3 independent biological experiments). **e-f,** 3CL significantly antagonized the innate immune response activate by MDA5 N terminal(CARD). HEK293T cells were transfected with NF-κB-Luc(**f**) or IFNβ-Luc(**g**), pRL-TK Renilla, and MDA5 CARD expression vector in the presence or absence of 3CL. Luciferase activity induced by MDA5 CARD alone used as the positive control and was set to 100%. Transactivation of the luciferase reporter was determined 24 h after transfection (n = 3 independent biological experiments) **(a-c, e-f)**. Means and standard deviations are presented. Statistical significance was determined by two-sided unpaired t-test, *** *p*<0.001; **** *p*<0.0001 **(a-c, e-f)**.

Figure. S3.

**
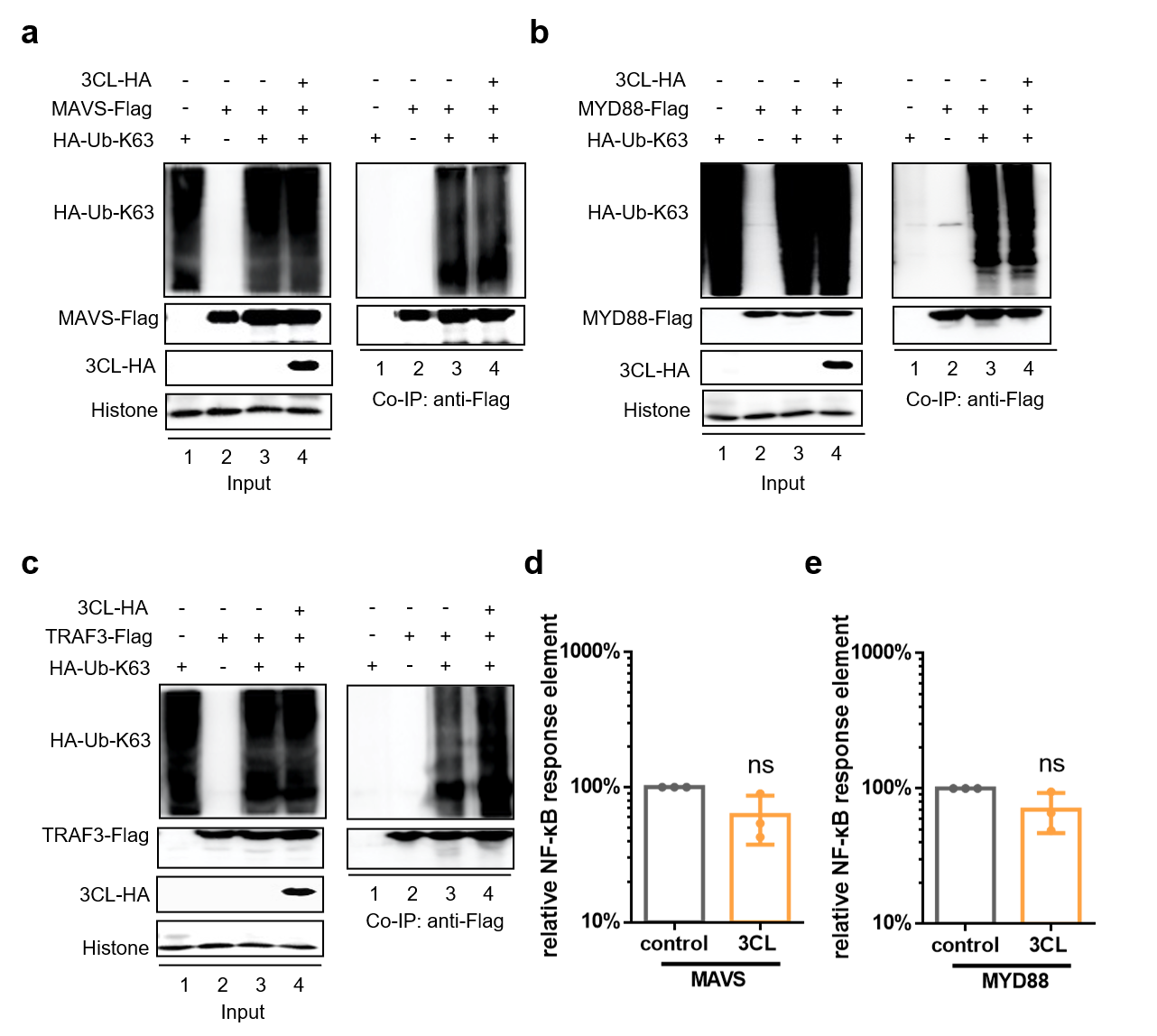
**

**Fig. S3 a-c,** Co-precipitation of MAVS **(a)**, MYD88 **(b)**, TRAF3 **(c)** with Ub-K63. HEK293T cells were transfected with Ub-K63 alone, MAVS or MYD88 or TRAF3 alone, or co-transfected MAVS or MYD88 or TRAF3 and Ub-K63, with or without 3CL. MG132 (20 μM) was added 12 h later. Cell lysates were prepared and reacted with anti-Flag antibody to detect Flag-MAVS **(a)**, Flag-MYD88 **(b)**, Flag-TRAF3 **(c)**, anti-HA antibody to detect HA-Ub-K63. Histone was used as the loading control. **d-e,** 3CL has no effect on MAVS or MYD88-stimulated NF-κB signaling. HEK293T cells were transfected with NF-κB-Luc, pRL-TK Renilla, and MAVS or MYD88 expression vectors in the presence or absence of 3CL. Luciferase activity induced by MAVS or MYD88 alone used as the positive control and was set to 100%. Transactivation of the luciferase reporter was determined 24 h after transfection (n = 3 independent biological experiments) . Means and standard deviations are presented. Statistical significance was determined by two-sided unpaired t-test, NS, no significance.

Figure. S4.
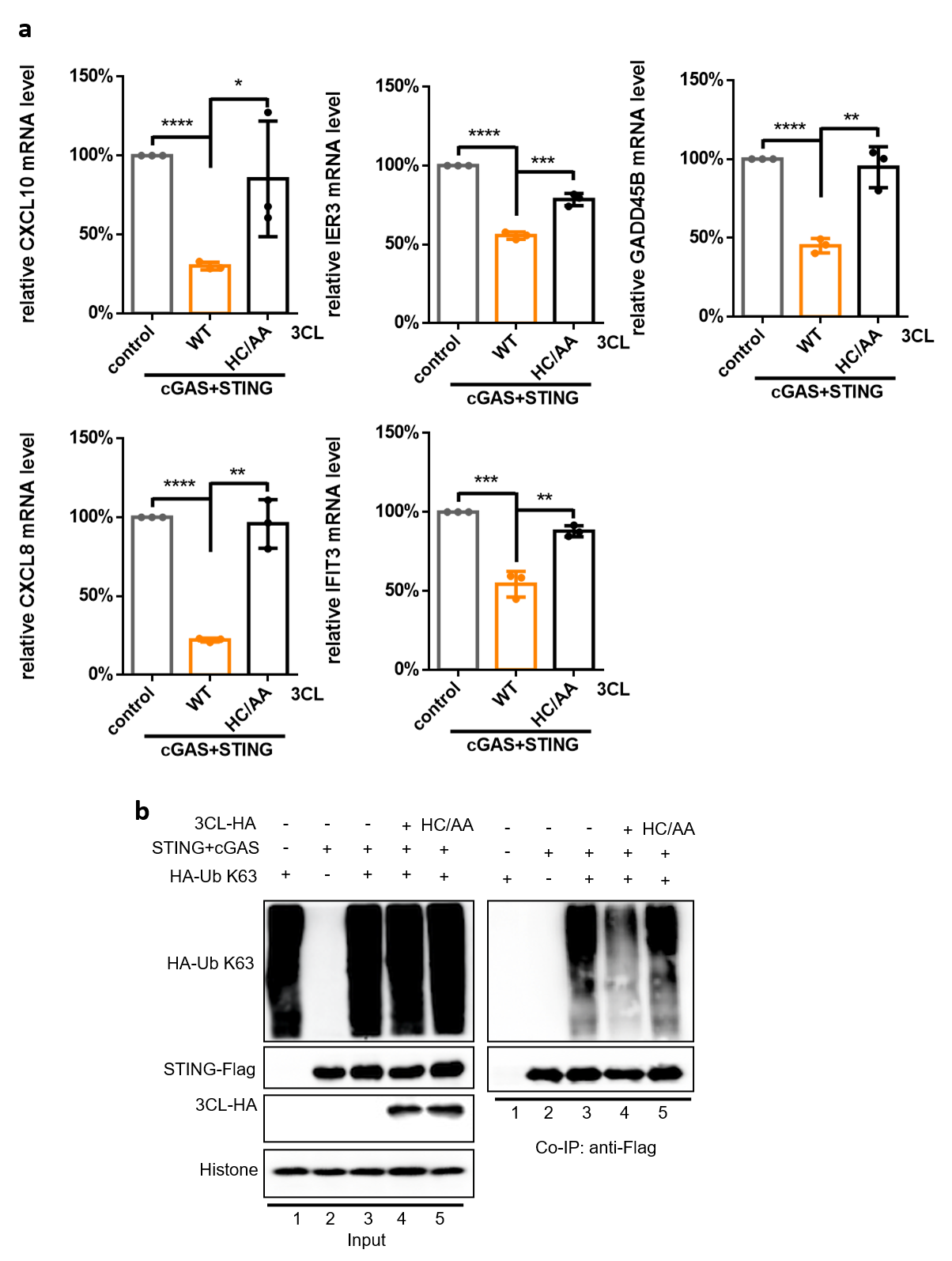


**Fig. S4 a,** The 3CL mutant HC/AA has a reduced ability to inhibit cGAS–STING-stimulated NF-κB-related genes. HEK293T cells were co-transfected with the cGAS-STING expression vectors in the presence or absence of 3CL or 3CL mutant HC/AA expression vectors. After 24 h, total RNA was prepared from the harvested cells and analyzed for the transcriptional levels of the indicated genes by RT-qPCR (n = 3 independent biological experiments). Human genes activated by cGAS-STING were used as the positive control and set to 100%. Means and standard deviations are presented. Statistical significance was determined by two-sided unpaired *t*-test, *p<0.05; **p<0.01; *** *p*<0.001; **** *p*<0.0001. **b,** The 3CL mutant HC/AA has no effect on K63-Ub modification of STING. HEK293T cells were transfected with Ub K63 alone, STING and cGAS alone, or co-transfected STING-cGAS and Ub K63, with 3CL or the 3CL HC/AA mutant. MG132 (20μM) was added 12 h later. Cell lysates were prepared and immunoprecipitated using anti-Flag beads 24 h after transfection. Precipitated samples were prepared and reacted with anti-Flag antibody to detect Flag-STING, anti-HA antibody to detect HA Ub-K63. Histone was used as the loading control.

Figure. S5.


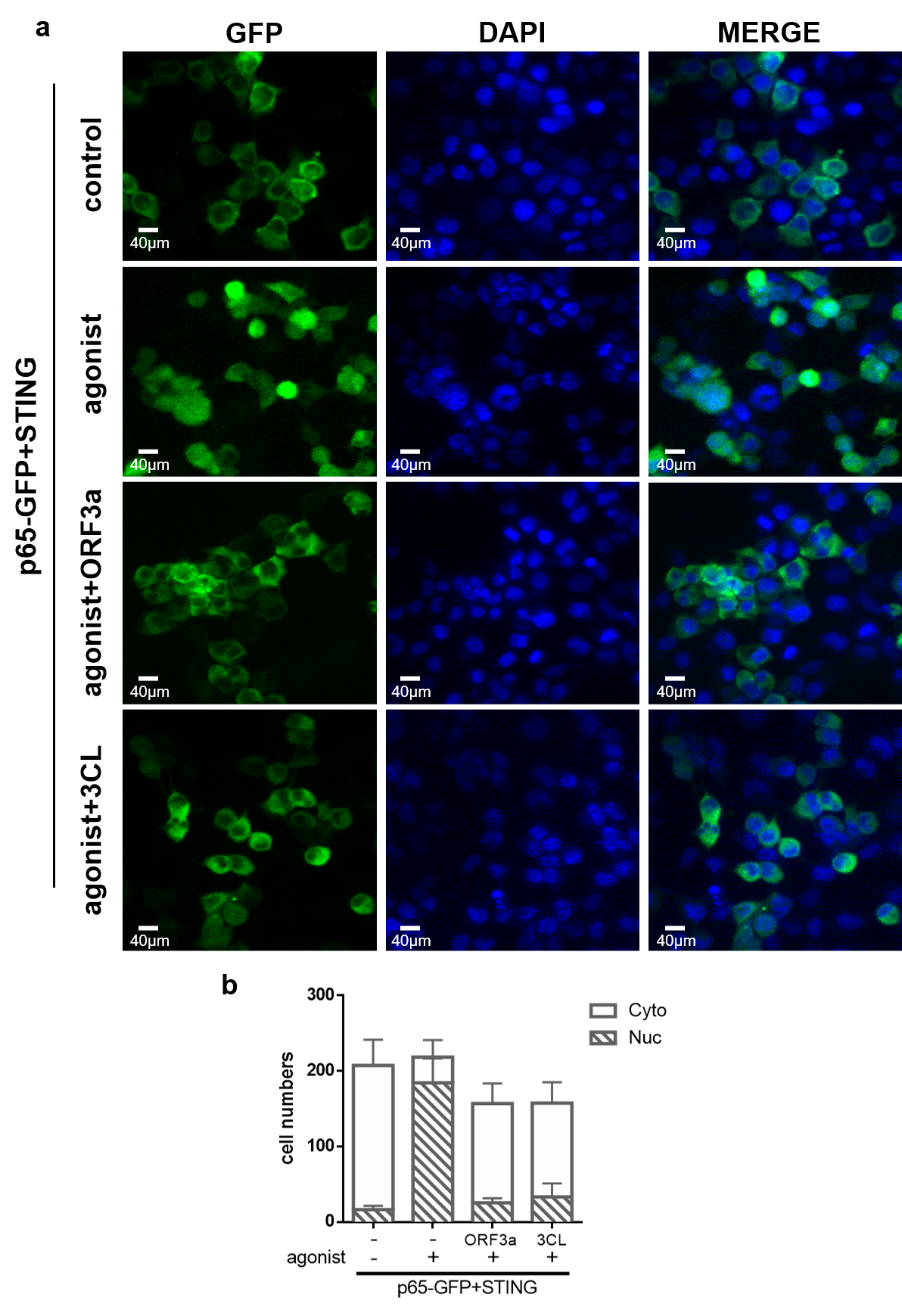


**Fig. S5 a,** HEK293T cells were co-transfected with p65-GFP, STING in the presence or absence of vectors expressing ORF3a or 3CL as indicated. 12 h after transfection, 1μM STING agonist-3 was added into medium for 6 h. DAPI staining was performed to show the nucleus.

**b,** The numbers of p65 nuclear translocation among total cells was shown in the bar graph. Representative statistics from n=6 independent biological experiments are shown. Means and standard deviations are presented.

**
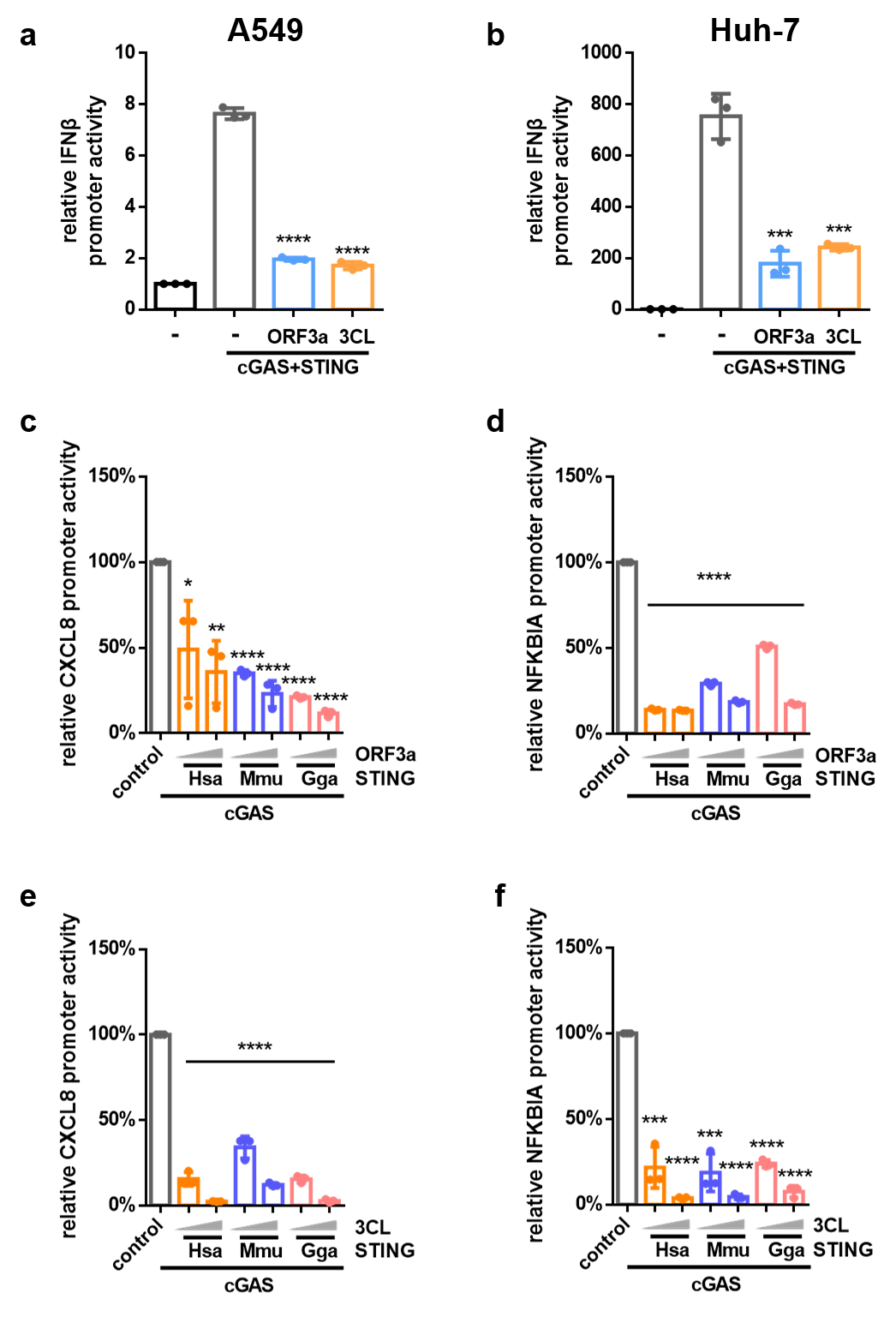
Figure. S6.**

**Fig. S6 a-b,** ORF3a and 3CL inhibit cGAS-STING triggered IFNβ promoter activity in A549 and Huh7 cell lines. A549 cells **(a)** or Huh7 cells **(b)** were co-transfected with IFNβ-Luc, pRL-TK Renilla and the cGAS and STING expression vectors in the presence or absence of vectors expressing ORF3a or 3CL as indicated. **c-f,** SARS-CoV-2 ORF3a and 3CL both inhibit vertebrate STING function in a dose-dependent manner. HEK293T cells were transfected with CXCL8-Luc **(c, e)**, or NFKBIA-Luc **(d, f)** and pRL-TK Renilla, together with STING-Flag and Myc-cGAS and increasing amounts of ORF3a **(c, d)** or 3CL **(e, f)** expression vector. At 24 h after transfection, the CXCL8, and NFKBIA promoter transactivation was analyzed by luciferase reporter assay (n = 3 independent biological replicates). Luciferase activity induced by cGAS-STING alone was used as the positive control and set to 100%. Means and standard deviations are presented. The statistical significance analyses were performed using a two-sided unpaired *t*-test, *p<0.05; **p<0.01; *** *p*<0.001; **** *p*<0.0001.

**Figure. S7.**


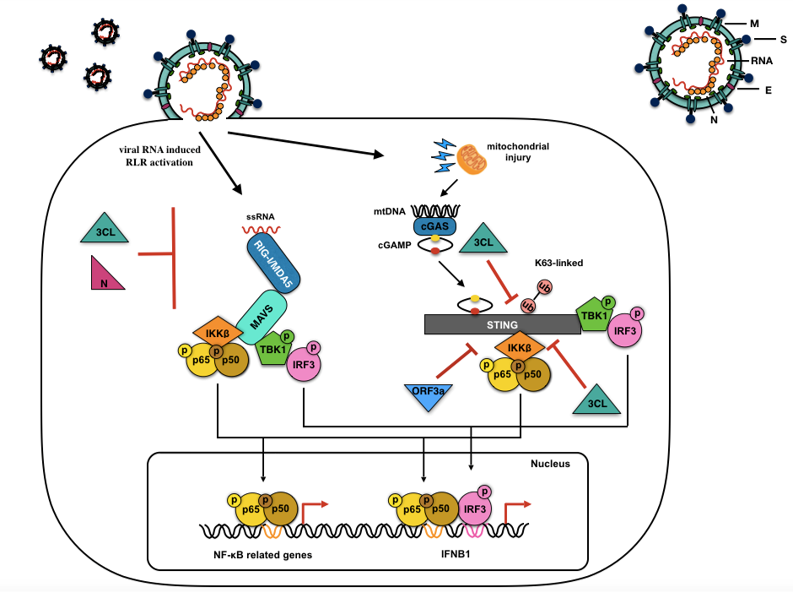


**Fig. S7** A model showing SARS-CoV-2-mediated suppression of RLR and cGAS-STING pathways. Viral RNAs activate the RLR pathway. Viral fusion and mitochondrial damage induced during viral infection activate the cGAS-STING pathway.

Table S1.

**Table 1 Antibodies Details**

| **Antibody (Clone name)** | **Species** | **Manufacturer** | **Catalogue #** | **Lot #** | **Dilution** | **PMID** |
| --- | --- | --- | --- | --- | --- | --- |
| **List of antibodies used for western blotting** | | | | | | |
| Histone (clone H3) | Rabbit | Genscript | A01502 | 18E001481 | 1:1000 | 23833181 |
| HA (16B12) | Mouse | Biolegend | 901514 | B272772 | 1:1000 | 30247716 |
| HA (polyconal) | Rabbit | invitrogen | 71-5500 | UK293079 | 1:1000 | 29343707 |
| Flag (clone M2) | Mouse | Sigma | F3165 | SLBJ46O71 | 1:1000 | 22426228 |
| Flag (polyconal) | Rabbit | Sigma | SAB4301135 | 851135535 | 1:1000 | 26935028 |
| Myc (4A6) | Mouse | Millipore | 05-724 | 3095953 | 1:1000 | 30247716 |
| p-IRF3 (clone 4D4G) | Rabbit | Cell Signal | 4947 | 13 | 1:500 | 31320712 |
| IκBα (L35A5) | Mouse | Cell Signal | 4814 | 17 | 1:1000 | 32733235 |
| IRF3(D6I4C) | Rabbit | Cell Signal | 11904S | 5 | 1:1000 | 32733001 |
| GAPHD (clone 1E6D9) | Mouse | Proteintech | 60004-1-Ig | 10003343 | 1:1000 | 31058095 |
| Alpha-tubulin (polyconal) | Mouse | Proteintech | 11224-1-AP | 10003004 | 1:1000 | 20197313 |
| IFIT3 (polyconal) | Rabbit | Proteintech | 15201-1-AP | 00064229 | 1:1000 | 27681138 |
| TBK1 (D1B4) | Rabbit | Cell Signal | 3504S | 4 | 1:1000 | 32209471 |
| p-TBK1 (S172) | Rabbit | Cell Signal | 5483S | 8 | 1:1000 | 32246052 |
| IKKβ(D30C6) | Rabbit | Cell Signal | 8943S | 4 | 1:1000 | 32268084 |
| p65 | Rabbit | Proteintech | 10745-1-AP | 057102 | 1:1000 | 28358376 |
| GFP | Rabbit | Proteintech | 50430-2-AP | 00078456 | 1:1000 | 19755120 |
| secondary antibodies goat anti-mouse (polyconal) | Goat | HuaBio | HA1006 | G180906 | 1:2500 | 28456659 |
| secondary antibodies goat anti-rabbit (polyconal) | Goat | HuaBio | HA1001 | G190128 | 1:2500 | 28456659 |
| anti-HA antibody-agarose conjugate | N/A | Roche | 610760 | 29732600 | N/A | 28424289 |
| anti-Flag M2 Affinity Gel (clone M2) | N/A | Sigma | A2220 | SLBT8835 | N/A | 17274760 |

| **Primer Name** | **Sequence** | **Gene accession number** |
| --- | --- | --- |
| IFNB1-F  IFNB1-R | CGCCGCATTGACCATCTA  GACATTAGCCAGGAGGTTCT | NM_002176 |
| CXCL8-F  CXCL8-R | CGGAAGGAACCATCTCACTGTG  AGAAATCAGGAAGGCTGCCAAG | NM_000584 |
| NFKBIA-F  NFKBIA-R | ACACCAGGTCAGGATTTTGC  GCTGATGTCAATGCTCAGGA | NM_020529 |
| CXCL10-F  CXCL10-R | GCCTCTCCCATCACTTCCCTAC  GAAGCAGGGTCAGAACATCCAC | NM_001565 |
| GADD45B-F  GADD45B-R | TCGGATTTTGCAATTTCTCC  GGATGAGCGTGAAGTGGATT | NM_015675 |
| IER3-F  IER3-R | GCCGCCTTCTAACTGTGACTC  GTCTCCGCTGTAGTGTTCTGAG | NM_003897 |
| GAPDH-F  GAPDH-R | ATGGGGAAGGTGAAGGTCG  GGGGTCATTGATGGCAACAATA | NM_002046 |
| IFIT1-F | GAAGCAGGCAATCACAGAAA | NM_001548.5 |
| IFIT1-R | TGAAACCGACCATAGTGGAA |  |
| IFIT2-F | AATGCCATTTCACCTGGAACTTG | NM_001547.5 |
| IFIT2-R | GTGATAGTAGACCCAGGCATA |  |
| IFIT3-F | AACTACGCCTGGGTCTACTATCACT | NM_001549.6 |
| IFIT3-R | ACACCTTCGCCCTTTCATTTC |  |
| TNFAIP3-F | AATCTTCCCCGGTCTCTGTT | NM_006290 |
| TNFAIP3-R | TACCCTTGGTGACCCTGAAG |  |

Table S2.

Table 2 Primers List
