# Supplementary material for: Unique and complementary suppression of cGAS-STING and RNA sensing- triggered innate immune responses by SARS-CoV-2 proteins
Source: Signal Transduct Target Ther. 2021 Mar 15;6:123. doi: 10.1038/s41392-021-00515-5 (PMC7958565; doi:10.1038/s41392-021-00515-5)
Supplement: Supplementary file 2 — Supplementary tables [file 41392_2021_515_MOESM2_ESM.pdf]

Supplementary Table 1 Antibodies Details

Antibody (Clone name)	Species	Manufacturer	Catalogue #	Lot #	Dilution	PMID
**List of antibodies used for western blotting**						
Histone (clone H3)	Rabbit	Genscript	A01502	18E001481	1:1000	23833181
HA (16B12)	Mouse	Biolegend	901514	B272772	1:1000	30247716
HA (polyconal)	Rabbit	invitrogen	71-5500	UK293079	1:1000	29343707
Flag (clone M2)	Mouse	Sigma	F3165	SLBJ46O71	1:1000	22426228
Flag (polyconal)	Rabbit	Sigma	SAB4301135	851135535	1:1000	26935028
Myc (4A6)	Mouse	Millipore	05-724	3095953	1:1000	30247716
p-IRF3 (clone 4D4G)	Rabbit	Cell Signal	4947	13	1:500	31320712
GAPHD (clone 1E6D9)	Mouse	Proteintech	60004-1-Ig	10003343	1:1000	31058095
Alpha-tubulin (polyconal)	Mouse	Proteintech	11224-1-AP	10003004	1:1000	20197313
IFIT3 (polyconal)	Rabbit	Proteintech	15201-1-AP	00064229	1:1000	27681138
TBK1 (D1B4)	Rabbit	Cell Signal	3504S	4	1:1000	32209471
p-TBK1 (S172)	Rabbit	Cell Signal	5483S	8	1:1000	32246052
IKKβ(D30C6)	Rabbit	Cell Signal	8943S	4	1:1000	32268084
p65	Rabbit	Proteintech	10745-1-AP	057102	1:1000	28358376
GFP	Rabbit	Proteintech	50430-2-AP	00078456	1:1000	19755120
secondary antibodies goat anti-mouse (polyconal)	Goat	HuaBio	HA1006	G180906	1:2500	28456659
secondary antibodies goat anti-rabbit (polyconal)	Goat	HuaBio	HA1001	G190128	1:2500	28456659
anti-HA antibody-agarose conjugate	N/A	Roche	610760	29732600	N/A	28424289
anti-Flag M2 Affinity Gel (clone M2)	N/A	Sigma	A2220	SLBT8835	N/A	17274760

Supplementary Table 2 Primers List

Primer Name	Sequence	Gene accession number
IFNB1-F	CGCCGCATTGACCATCTA	NM_002176
IFNB1-R	GACATTAGCCAGGAGGTTCT	
CXCL8-F	CGGAAGGAACCATCTCACTGTG	NM_000584
CXCL8-R	AGAAATCAGGAAGGCTGCCAAG	
NFKBIA-F	ACACCAGGTCAGGATTTTGC	NM_020529
NFKBIA-R	GCTGATGTCAATGCTCAGGA	
CXCL10-F	GCCTCTCCCATCACTTCCCTAC	NM_001565
CXCL10-R	GAAGCAGGGTCAGAACATCCAC	
GADD45B-F	TCGGATTTTGCAATTTCTCC	NM_015675
GADD45B-R	GGATGAGCGTGAAGTGGATT	
IER3-F	GCCGCCTTCTAACTGTGACTC	NM_003897
IER3-R	GTCTCCGCTGTAGTGTTCTGAG	
GAPDH-F	ATGGGGAAGGTGAAGGTCG	NM_002046
GAPDH-R	GGGGTCATTGATGGCAACAATA	
IFIT1-F	GAAGCAGGCAATCACAGAAA	NM_001548.5
IFIT1-R	TGAAACCGACCATAGTGGAA	
IFIT2-F	AATGCCATTTCACCTGGAACTTG	NM_001547.5
IFIT2-R	GTGATAGTAGACCCAGGCATA	
IFIT3-F	AACTACGCCTGGGTCTACTATCACT	NM_001549.6
IFIT3-R	ACACCTTCGCCCTTTCATTTC	
TNFAIP3-F	AATCTTCCCCGGTCTCTGTT	NM_006290
TNFAIP3-R	TACCCTTGGTGACCCTGAAG	
